# Six years follow-up of type 1 retinopathy of prematurity (ROP) treated with intravitreal injection of ranibizumab and bevacizumab: Long-Term Outcomes of intravitreal injection in Type 1 ROP

**DOI:** 10.1097/MD.0000000000039251

**Published:** 2024-08-09

**Authors:** Hsiao-Fan Tung, Yi-Ling Chen, Yen-Chih Chen, Shin-Lin Chiu, San-Ni Chen

**Affiliations:** a Department of Ophthalmology, China Medical University Hospital, Taichung, Taiwan; b Department of Surgery, Clinical Research Center, Changhua Christian Hospital, Changhua, Taiwan; c Department of Ophthalmology, Changhua Christian Hospital, Changhua, Taiwan; d Department of Nursing, College of Nursing and Health Sciences, Da-Yeh University, Changhua, Taiwan; e Department of Post-Baccalaureate Medicine, College of Medicine, National Chung Hsing University, Taichung, Taiwan; f Department of Optometry, Da-Yeh University, Changhua, Taiwan; g Department of Medicine, School of Medicine, Chung-Shan Medical University, Taichung, Taiwan.

**Keywords:** retinopathy of prematurity, intravitreal injection, ranibizumab, bevacizumab

## Abstract

To investigate biometric and refractive results in patients with type 1 retinopathy of prematurity (ROP) treated by intravitreal injection (IVI) of ranibizumab (R) and bevacizumab (B) at the corrected age of 6. This is a single-center retrospective study. Infants diagnosed with type 1 ROP and treated with IVI of either R or B as the primary therapy were included. Data on axial length, anterior chamber depth (ACD), and lens thickness (LT) were obtained using A-scan ultrasound. Cycloplegic refraction, keratometry (K), and best-corrected visual acuity were also documented. Additionally, optical coherence tomography angiography was performed to assess the foveal avascular zone and the density of superficial and deep vessels. We analyzed the structural and functional differences between the 2 groups and compared them with findings from a previous study conducted when these children were between the ages of 1 and 3. The study included 60 eyes from 34 patients, with 34 eyes receiving B and 26 eyes receiving R injections for ROP. In biometric outcomes, there was still a deeper ACD (3.36 ± 0.24 mm in the B group; 3.52 ± 0.21 mm in the R group) and thinner LT (3.63 ± 0.16 mm in the B group; 3.53 ± 0.12 mm in the R group) in the R group, as previously reported at the age of 3. In the refractive aspect, the eyes treated with B had higher myopia at the ages of 1 and 3; however, at the age of 6, refractive errors did not differ significantly between the 2 groups. At the corrected age of 6, the eyes treated with IVI of R were associated with deeper ACD and thinner LT. Interestingly, the emmetropization process resulted in a similar incidence of high myopia at the age of 6, which was different from the outcomes observed at younger ages.

## 1. Introduction

Retinopathy of prematurity (ROP) is an immature retinal disease that results in retinal neovascularization, threatening visual function in childhood. According to the current consensus, except for stage 4 and stage 5 ROP, surgical treatments are suggested for type 1 ROP, which includes Zone I with plus disease; Zone I, stage 3 without plus disease; and Zone II, stage 2 or 3 with plus disease. Conventionally, cryopexy and laser therapy for the avascular retina were the standard treatments. With a better understanding of intravitreal injection (IVI) of anti-vascular endothelial growth factor (Anti-VEGF), it has been reported to be effective in treating type 1 ROP.^[1–3]^ In addition, better biometric and refractive outcomes have been found in eyes treated with IVI anti-VEGF compared to laser therapy. The structural development of the fovea was also more favorable with IVI compared to laser treatment.^[4–6]^ Among the anti-VEGF treatments, bevacizumab (B) was first applied for type 1 ROP, followed by ranibizumab (R), which was reported to have fewer systemic complications in adults but is more expensive than B.^[7]^

In 2018, Chen e al^[8]^ compared the biometric and refractive outcomes of IVI of R and B for type 1 ROP. The study found a shallower ACD and a higher prevalence of refractive error in patients treated with IVI of B at the corrected age of 3. In this study, we are interested in the long-term outcomes in these children, extending the follow-up period to the age of 6.

## 2. Material and methods

This is a single-center retrospective study conducted at Changhua Christian Hospital, Changhua City, Taiwan. The study was approved by the Institutional Review Board of Changhua Christian Hospital, and all research was performed in accordance with relevant guidelines and regulations. This study was conducted according to the tenets of the Declaration of Helsinki. Informed consent was legally obtained from the parents before the surgical procedures were performed. Infants diagnosed with type 1 ROP and treated with IVI of either R or B as the primary therapy at Changhua Christian Hospital from April 2010 to February 2015 were enrolled. Those who received cryopexy and laser treatments were excluded. The same criteria followed by the indications for laser treatments in the Early Treatment for ROP study were applied.^[9]^ Before the surgery, the risks, complications, and efficacy of laser and IVI of anti-VEGF were thoroughly explained to the parents. The differences between R and B were also fully disclosed to the parents, including the possibility of systemic suppression and the costs.

We collected data on birth history, including birth body weight (BBW), gestational age (GA), and postmenstrual age (PMA) at the time of treatment. Additionally, we recorded the stage and zone of ROP and the corrected age at each visit. Best-corrected visual acuity (BCVA), axial length (AL), ACD, LT, and cycloplegic refraction, which included spherical equivalent (SE) and cylinder (C), were checked at least once a year. AL, ACD, and LT were measured by A-scan ultrasound (model A-1500; Sonomed, Lake Success, NY, USA). Cycloplegic refraction and keratometry (K) were obtained using the kerato-refractometer (Topcon KR-8100, Tokyo, Japan). We defined and classified the refractive error according to Chen method^[8]^; as follows: high myopia (SE ≤ −5D), low myopia (−5D < SE ≤ −1D), emmetropia (−1D < SE < +1D), low hyperopia (+1D ≤ SE ≤ +4D), and high hyperopia (SE > +4D). Additionally, optical coherence tomography angiography (OCT-A) was performed with the Avanti RTVue XR system (Optovue, Fremont, CA). Using macular protocols for 6 mm × 6 mm and 3 mm × 3 mm, we obtained automated data on the size and area of the foveal avascular zone (FAZ) and the density of superficial and deep vessels. All images and data were rechecked by 2 researchers. If the image quality was poor or other errors were found (e.g., too much artifact to identify the foveal structure), it was excluded. Patients without complete medical records were also excluded.

The IVI procedure was performed under topical anesthesia with 0.5% proparacaine hydrochloride. The dosage of the injected anti-VEGF was 0.625 mg/0.025 mL for B or 0.25 mg/0.025 mL for R. Weekly follow-ups were arranged to monitor the response to IVI anti-VEGF until the regression of ROP was observed. After stabilization, patients routinely visited our clinic for regular ocular examinations until at least 6 years of corrected age.

Statistical analysis was performed using SPSS software, version 23.5. The logarithm of the minimal angle of resolution (logMAR), converted from the Snellen BCVA, was used for statistical analysis. The Wilcoxon Signed-Rank Test was used to compare differences between the 2 groups, including baseline data (BBW, GA, PMA at IVI, corrected age), biometric conditions (AL, ACD, LT), and refractive status (SE, C). Chi-square tests were used to analyze the prevalence of different classes of refractive error in the 2 groups. Univariate linear regression and multivariate linear analysis were applied to evaluate the correlation between BCVA and all factors. A *P* value < .05 was considered statistically significant in all analyses.

## 3. Results

A total of 60 eyes from 34 patients were enrolled. Among them, 34 eyes in 20 patients were treated with IVI of B, and the remaining 26 eyes in 14 patients were treated with IVI of R. In the B group, a patient with initially bilateral zone 1, stage 3 ROP with plus sign received a second injection due to zone 2, stage 2 with plus sign being found in both eyes weeks after the first injection. In the R group, 1 patient with bilateral zone 1, stage 3 ROP also underwent a secondary R injection due to persistent type 1 ROP in both eyes several weeks later. There was no statistical difference (*P* = .605) in the number of injections between the 2 groups. Table 1 shows the baseline characteristics, birth data, and ROP status of all subjects.

**Table 1:** Baseline characteristics, birth data and retinopathy of prematurity status of all subjects.

	Bevacizumab	Ranibizumab	*P* value
Patient number^a^	20	14	
Eyes^a^	34	26	
Female: Male^a^	12: 8	6: 8	.084
Age^a^	6.00 ± 0.79	5.86 ± 1.03	.380
GA (wks)^a^	27.48 ± 3.30	26.93 ± 2.30	.891
BBW(g)^a^	891.48 ± 207.33	826.64 ± 250.00	.346
ROP zone^b^			
I	2 (6%)	2 (8%)	.883
II	32 (94%)	24 (92%)	
ROP stage^b^			
2	0 (0%)	2 (8%)	.564
3	34 (100%)	24 (92%)	

a Wilcoxon Signed-Rank Test.

b Chi-square test.

Table 2 reveals the biometric and refractive outcomes in our patients. Better BCVA was found in patients who received intravitreal injection of B at the corrected age of 6. Additionally, significantly deeper ACD (B: 3.36 ± 0.24 mm; R: 3.52 ± 0.21 mm; *P* value < .05) and thinner LT (B: 3.63 ± 0.16 mm; R: 3.53 ± 0.12 mm; *P* value < .05) were noted in the R group. No statistical differences were found in AL, K, and refractive outcomes between the 2 groups. Similarly, no differences were noted in foveal microvasculature between the 2 groups.

**Table 2:** Biometric, refractive, and foveal microvasculature outcomes in the 2 groups.

	Bevacizumab	Ranibizumab	*P* value
logMAR VA	0.03 ± 0.09	0.09 ± 0.12	.007*
AXL (mm)	22.45 ± 0.92	22.26 ± 0.76	.623
ACD (mm)	3.36 ± 0.24	3.52 ± 0.21	.013*
LT (mm)	3.63 ± 0.16	3.53 ± 0.12	.042*
K (diopters)	44.50 ± 1.18	45.33 ± 2.31	.266
SE (diopters)	0.32 ± 2.53	0.32 ± 2.01	.398
C (diopters)	1.17 ± 1.23	1.13 ± 0.99	.691
FAZ (mm^2^)	0.11 ± 0.08	0.12 ± 0.08	.765
Superficial VD (%)	47.25 ± 3.76	46.32 ± 3.57	.239
Deep VD (%)	49.60 ± 5.75	50.60 ± 3.61	.571

Wilcoxon Signed-Rank Test.

ACD = anterior chamber depth, AXL = axial length, C = cylinder, FAZ = foveal avascular zone, K = keratometry, LT = lens thickness, SE = spherical equivalent, VA = visual acuity, VD = vessel density.

* Statistically significant (*P* value < .05) in comparison of the 2 groups.

When considering refractive errors, no patient presented with high hyperopia (≥+4D) in either group. The highest diopter of hyperopia in our data was 3.75 D in a patient treated with IVI of B. When we categorized the refractive errors as shown in Table 3, there were no statistically significant differences between the groups.

**Table 3:** Refractive errors of the type 1 retinopathy of prematurity patient treated with bevacizumab or ranibizumab at the corrected age of 6.

	Bevacizumab	Ranibizumab	*P* value
High myopia (≤−5.0 D)	2 (06%)	2 (08%)	.593
Myopia (≤−1.0 D)	5 (15%)	4 (15%)	>.999
Hyperopia (>+1.0 D)	18 (53%)	9 (35%)	.055
Ametropia (>+1 D or <−1 D)	23 (68%)	13 (50%)	.098
High astigmatism	8 (24%)	4 (15%)	.150

Chi-square test.

Linear regression was performed to analyze the correlation between BCVA and all parameters. Simple regression showed that patients with deeper ACD, lower degree of K, higher SE, receiving IVI of B, and initially presenting a greater ROP zone tended to have better BCVA. Further multiple regression analysis, including the above factors, confirmed that deeper ACD, higher SE, the choice of B, and the initial presence of a greater ROP zone were significantly correlated with better BCVA (Table 4).

**Table 4:** Correlation between visual acuity and the biometry, refractory, ocular characteristics, and the kinds of anti-vascular endothelial growth factor.

	Linear regression with logMAR VA			
	Simple regression analysis		Multiple regression analysis	
	*r*	*P*	*r*	*P*
AXL	0.094	.473		
ACD	−0.297	.026*	−0.305	.014*
LT	−0.044	.752		
K	0.264	.042*	−0.141	.258
SE	−0.484	<.001*	−0.500	<.001*
C	0.219	.093		
Anti-VEGF^a^	0.274	.034*	0.341	.006*
ROP zone	−0.389	.002*	−0.474	.013*
ROP stage	0.095	.469		

ACD = anterior chamber depth, Anti-VEGF = anti-vascular endothelial growth factor, AXL = axial length, C = cylinder, K = keratometry, LT = lens thickness, SE = spherical equivalent, VA = visual acuity.

* Statistically significant (*P* value < .05).

a The definition in analyze: bevacizumab = 1, ranibizumab = 2.

## 4. Discussion

In this study, we compared biometric and refractive outcomes in patients with type 1 ROP who were treated with IVI of B or R at the corrected age of 6 years. The BCVA was better in the B group. From the biometric data, we found deeper ACD and thinner LT in patients treated with R. However, there were no differences between the 2 groups in refractive and foveal microvascular aspects. Though there was no statistically significant difference in AL, the mean AL was longer in the B group. This may mitigate the other factors (shallower ACD and thicker LT), resulting in similar refractive outcomes in the 2 groups.

Previous studies have reported different outcomes for these 2 anti-VEGF treatments at the ages of 1 and 3.^[8,10]^ The children in our study were almost the same participants as those published by Chen et al.^[8]^ Interestingly, the eyes treated with B had a higher myopic rate at the ages of 1 and 3. However, the incidence of myopia did not differ at the age of 6. When checking the biometric data, deeper ACD and thinner LT were still found in the R group at the age of 3.

Several hypotheses could explain this situation. First, pharmacological and dosage differences: B has a longer intraocular half-life compared to R. This could influence the duration and intensity of VEGF inhibition in the developing eye, potentially affecting refractive outcomes differently at various developmental stages. The longer-lasting effect of B might initially delay the emmetropization process (the natural correction of refractive error) more than R. However, as our results at the age of 6 suggest, this delay may not persist into later childhood, aligning with the natural tapering of myopic progression as the eye grows and matures. Moreover, differences in anterior chamber depth (ACD) and lens thickness (LT) between the 2 groups at earlier ages are closely linked to refractive development. The initial greater suppression of eye growth by B could have temporarily promoted myopia, which later normalized due to compensatory growth mechanisms as the children aged.

The above findings disclosed that emmetropization occurred and brought about similar refractive errors when the children grew up to the age of 6. In other words, although the development of the anterior segment was more ideal in the R group, there was no impact on the refractive outcome. Notably, the BCVA at the age of 6 was better in the B group compared to the R group. Our study’s results reveal that the efficacy and refractive outcomes of B are not less than those of R. Clinically, the cost of R is more expensive than B, which plays an important role in decision-making, especially for patients with financial constraints. There are some limitations in our study, including its retrospective nature and small database. Further prospective studies with larger patient populations will be needed.

In conclusion, at the corrected age of 6, the eyes treated with IVI of R were associated with deeper ACD and thinner LT. The emmetropization process brought a similar incidence of high myopia at the age of 6, differing from the outcomes observed at younger ages.

## Author contributions

**Conceptualization:** Hsiao-Fan Tung, San-Ni Chen.

**Data curation:** Hsiao-Fan Tung, Yi-Ling Chen, Yen-Chih Chen.

**Resources:** Hsiao-Fan Tung, San-Ni Chen, Shin-Lin Chiu.

**Writing – original draft:** Hsiao-Fan Tung.

**Project administration:** San-Ni Chen.

**Writing – review & editing:** San-Ni Chen.

**Formal analysis:** Yi-Ling Chen.

**Methodology:** Yi-Ling Chen, Yen-Chih Chen.

**Software:** Yi-Ling Chen.

**Investigation:** Yen-Chih Chen.

**Supervision:** Shin-Lin Chiu.

**Validation:** Shin-Lin Chiu.

**Visualization:** Shin-Lin Chiu.
